# Evaluation of Turmeric Powder Adulterated with Metanil Yellow Using FT-Raman and FT-IR Spectroscopy

**DOI:** 10.3390/foods5020036

**Published:** 2016-05-17

**Authors:** Sagar Dhakal, Kuanglin Chao, Walter Schmidt, Jianwei Qin, Moon Kim, Diane Chan

**Affiliations:** United States Department of Agriculture/Agricultural Research Service, Environmental Microbial and Food Safety Laboratory, Bldg. 303, Beltsville Agricultural Research Center East, 10300 Baltimore Ave., Beltsville, MD 20705-2350, USA; sagar.dhakal@ars.usda.gov (S.D.); walter.schmidt@ars.usda.gov (W.S.); jianwei.qin@ars.usda.gov (J.Q.); klchao@hotmail.com (M.K.); diane.chan@ars.usda.gov (D.C.)

**Keywords:** FT-Raman, FT-IR, turmeric powder, metanil yellow, quantitative analysis

## Abstract

Turmeric powder (*Curcuma longa* L.) is valued both for its medicinal properties and for its popular culinary use, such as being a component in curry powder. Due to its high demand in international trade, turmeric powder has been subject to economically driven, hazardous chemical adulteration. This study utilized Fourier Transform-Raman (FT-Raman) and Fourier Transform-Infra Red (FT-IR) spectroscopy as separate but complementary methods for detecting metanil yellow adulteration of turmeric powder. Sample mixtures of turmeric powder and metanil yellow were prepared at concentrations of 30%, 25%, 20%, 15%, 10%, 5%, 1%, and 0.01% (*w/w*). FT-Raman and FT-IR spectra were acquired for these mixture samples as well as for pure samples of turmeric powder and metanil yellow. Spectral analysis showed that the FT-IR method in this study could detect the metanil yellow at the 5% concentration, while the FT-Raman method appeared to be more sensitive and could detect the metanil yellow at the 1% concentration. Relationships between metanil yellow spectral peak intensities and metanil yellow concentration were established using representative peaks at FT-Raman 1406 cm^−1^ and FT-IR 1140 cm^−1^ with correlation coefficients of 0.93 and 0.95, respectively.

## 1. Introduction

Turmeric (*Curuma long* L.) is a herbaceous root commonly used for food seasoning as well as for medicinal purposes. Turmeric has a long history of medicinal use in Asian countries [1,2] and is used in root, oil, and powder forms. Its medicinal value is mainly due to its content of curcumin (diferuloyol methane) [3,4,5,6,7], with attributed medical properties including anti-inflammatory [8,9,10,11,12], anticarcinogenic [13,14,15,16], antioxidant [17,18,19], and wound-healing [20,21,22] effects. Curcumin has also been reported to have promise for development of therapies for Alzheimer’s disease [23].

The curcumin content in turmeric varies. Studies have shown that factors such as nutrient and acidity content in soil [24,25], fertilizer, soil type and cultivar [26,27,28] affects the curcumin content in turmeric. Reported curcumin concentrations in turmeric range from 0.3% to 8.6% [3,8,29,30,31]. Curcumin is isolated from turmeric for medicinal and cosmetic purposes. Although whole, dried, or fresh turmeric are typically free of contamination, turmeric powder can be adulterated with different chemical powders used as substitutes for curcumin [32]. Studies have reported the mixing of *Curcuma zedoaria*, a wild relative of turmeric, into turmeric powder due to its close resemblance with turmeric [32,33]. Similarly, metanil yellow (C_18_H_14_N_3_NaO_3_S) is a toxic azo dye that has been added to turmeric powder to mimic the appearance of curcumin [34,35,36] when the actual curcumin content is low [37]. Toxicologically, metanil yellow is classified as a CII category substance by the Joint FAO/WHO Expert committee on Food Additives, and it implies that it is a compound for which virtually no information on long-term toxicity is available [38]. Toxicity is categorized into four classes according to the toxicity of chemicals, where Category I chemicals are the most toxic and poisonous, and Category IV chemicals are least toxic and poisonous. Studies on rats show that long term consumption of metanil yellow causes neurotoxicity [39], hepatocellular carcinoma [40,41,42], tumor development [43], deleterious effect on gastric mucin [44], and lymphocytic leukemia [45].

A variety of conventional methods have been effectively used for detection of metanil yellow in food stuffs. Ion-pair liquid chromatography detected with 99% linearity the presence of azo dyes, such as metanil yellow, in the range of 0.05 ppm to 10 ppm in food [46]. Similarly other methods such as high performance liquid chromatography-electrospray ionization tandem mass spectrometry [47], high performance capillary electrophoresis [48], and micellar chromatographic method [49] have been used for detection of metanil yellow and other dyes in food and beverages. Despite their high accuracies and satisfactory detection limits, these conventional methods are limited in practicality due to their operational complexity and sample-destructive nature. In contrast, the relative simplicity of optical methods has driven their increasing use for safety and quality detection of foods and food products [50,51,52,53,54]. This study made use of FT-Raman and FT-IR spectroscopy for evaluation of metanil yellow in turmeric powder. Although FT-IR and FT-Raman spectroscopy have not been previously reported for detection of metanil yellow adulteration in food, these spectroscopy methods have been widely used for detection of other food adulterants. Lohumi *et al*. (2014) used FT-NIR and FT-IR spectroscopy to detect starch adulteration in onion powder [55]. Similarly, studies have reported analysis of starch-adulterated garlic powder [56] and detection of palm oil in extra virgin olive oil [57] using FT-IR spectroscopy, detection of hazelnut oil in olive oil using FT-Raman and FT-MIR spectroscopy [58], and adulteration of virgin olive oil using FT-Raman spectroscopy [59]. FT-IR and FT-Raman spectroscopy are also used for detection of chemical contamination in spices. Schulz *et al*. (2003) applied FT-IR and FT-Raman spectroscopy for detection of carcinogenic compounds (methyleugenol and estragole) in basil [60]. Similarly, FT-IR was applied for detection and quantification of food colorants such as Tartrazine, Sunset yellow, Szorubine, Quinoline-yellow, Allura red, and Sudan II in saffron [61].

This study presents a comprehensive study of FT-Raman and FT-IR spectra of metanil yellow, turmeric powder, and turmeric adulterated with metanil yellow at different concentrations. The primary objectives of this study are to:

1 Study the FT-Raman and FT-IR spectra of metanil yellow and turmeric powder
2 Identify the FT-Raman and FT-IR spectral fingerprint of metanil yellow to distinguish it from the spectral signal of turmeric powder
3 Evaluate turmeric adulterated with metanil yellow at different concentrations and determine the minimum concentrations detectable by the FT-Raman and FT-IR methods
4 Quantitatively evaluate the concentration of metanil yellow mixed with turmeric using FT-Raman and FT-IR methods

## 2. Materials and Methods

### 2.1. Sample Preparation

Metanil yellow (70% dye, Aldrich, Carson City, NV, USA), organic turmeric powder (Norway, IA, USA), and de-ionized water (Thermo Scientific, St. Louis, MO, USA) were used to prepare sample mixtures. The high solubility of metanil yellow in water was used to obtain nicely mixed samples by first adding 0.5 g of de-ionized water at room temperature to 0.01 g of metanil yellow, followed by 3 min of vortex mixing. An appropriate amount of turmeric powder was then added to the metanil yellow to achieve the targeted sample concentration. Samples were prepared in this way for eight concentrations of metanil yellow: 30%, 25%, 20%, 15%, 10%, 5%, 1%, and 0.01% (*w/w*). Three replicates of each concentration were prepared for FT-Raman analysis, and three replicates were similarly prepared for FT-IR analysis.

### 2.2. FT-Raman and FT-IR Spectral Acquisition

A Fourier Transform spectroscopy (Thermo Scientific, Madison, WI, USA) that consisted of FT-IR module (Nicolet 6700, Thermo Scientific, Madison, WI, USA) and FT-Raman module (NXR-FT Raman module, Thermo Scientific, Madison, WI, USA) was utilized for collection of spectral data from samples. Each spectroscopy was designed with a separate operation chamber. The FT-IR module consisted of a triglycine sulfate (DTGS) detector with KBr beam splitter for collection of sample spectra in the spectral range of 650 cm^−1^ to 4000 cm^−1^. The attenuated total reflection (ATR) technique was utilized for FT-IR spectral collection. A small amount of the sample was placed on the Germanium crystal of the ATR device pressurized by pointed tip to ensure uniformity in surface area of contact between the Germanium crystal and sample. A background spectra was first acquired with the empty Germanium crystal prior to spectral collection of the sample. The crystal plate and pointed pressure tip of the ATR device was cleaned thoroughly using cotton soaked with methanol after spectral acquisition of each sample. An average of 32 successive scans at 4 cm^−1^ intervals were acquired and saved (“comma separated values” format) for further analysis. The FT-IR spectral signal of three replicate samples were acquired at each concentration level.

FT-Raman spectroscopy utilized a Germanium detector and CaF_2_ beam splitter for spectral acquisition in the spectral range of 100 cm^−1^ to 3700 cm^−1^. The Germanium detector was cooled by liquid nitrogen to reduce dark current noise and enhance signal-to-noise ratio. A Nuclear Magnetic Resonance (NMR) tube sampling accessory was used to hold the NMR tube in the FT-Raman compartment. Samples were put in NMR tubes (5 mm diameter, 50 mm length) to fill approximately one fourth of each tube’s length. Each sample tube was clamped in the NMR sampling accessory inside the FT-Raman compartment one at a time. The accessory was designed to move in horizontal and vertical directions using two stepper motors. Horizontal movement ensured the alignment of the NMR tube with the laser, and vertical movement was used to achieve the best laser focus. A laser source of 1064 nm (laser power 1 W) was used for sample excitation. An average of 32 successive scans at 8 cm^−1^ intervals were acquired and saved (“comma separated values” format) for further analysis. The FT-Raman spectral signal of three replicate samples were acquired at each concentration level.

The overall system was controlled by OMNIC software provided by Thermo Scientific (Madison, WI, USA). Prior to collection of the spectral signal, OMNIC software was adjusted to operate in FT-IR module (for FT-IR spectral collection) and FT-Raman module (for FT-Raman spectral collection).

### 2.3. Processing of Spectra Signal

Pre-treatments for the FT-Raman and FT-IR spectra were performed using Matlab (MathWorks, Natick, MA, USA). The raw FT-Raman spectral data were first treated for noise removal by applying a Savitzky-Golay filter, fitting a 5th order polynomial with 3-point interval by linear least squares method. Polynomial curve fitting was then used for removal of the fluorescence background, which is important even though the FT-Raman technique utilizing 1064 cm^−1^ is reportedly affected less by fluorescence background compared to other Raman techniques. Although other methods are available, the polynomial fitting method was utilized in this study because of its simplicity [50,51]: a polynomial curve is generated to fit the background fluorescence signal, and this curve is subtracted from the Raman spectra to remove the fluorescence background from the Raman spectral data. A 6th order polynomial curve was generated to fit the background fluorescence signal in the range of 850 cm^−1^ to 2800 cm^−1^. Since all the spectral peaks of metanil yellow, turmeric, and mixture samples were inside this spectral range, all further analysis used only the corrected FT-Raman data from this region.

Similarly, the raw FT-IR spectra were first pre-treated for noise removal by a Savitzky-Golay filter using a 5th order polynomial with 3-point interval to fit the spectral signal by linear least squares method. Multiplicative Scatter Correction (MSC) was utilized to remove the baseline drift from the FT-IR spectra of mixture samples.

Two spectral analysis methods were compared for evaluating metanil yellow concentration in the mixture samples. First, the spectral peak intensity of metanil yellow after data-pretreatment was correlated with the actual concentration of metanil yellow in the mixture sample. The second approach was a band ratio method, whereby a ratio was obtained of the intensity at a peak representative of metanil yellow against the intensity of a neutral spectral band, and the ratio was correlated with metanil yellow concentration in the sample mixtures. The neutral band was selected as being one not exhibiting any sharp peaks due to metanil yellow, turmeric powder, or any other chemical constituents. Each of these two methods was applied using the FT-Raman spectral measurements and, separately, using the FT-IR spectral measurements.

## 3. Results and Discussion

### 3.1. Spectral Interpretation

**Figure 1:**
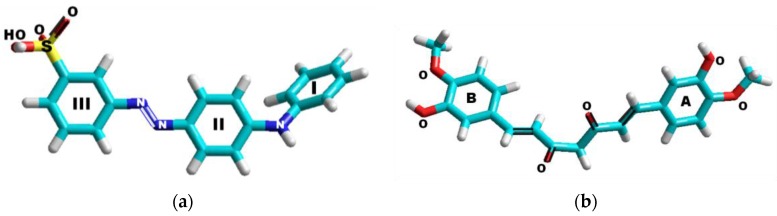
Chemical structure of: Metanil yellow (**a**), and Curcumin/Turmeric (**b**).

The comparable degree of extended conjugation in both metanil yellow and curcumin/turmeric is responsible for the similar yellow color present in each chemical substance. However, the chemical composition in each is very different. Metanil yellow contains three nitrogen atoms (N=N and –NH) and one sulfate (SO_3_^−^) group; it contains no methylene (CH_2_) or methyl (CH_3_) groups, and no oxygens except for those on the sulfate site (Figure 1a). Curcumin/Turmeric contains six oxygen atoms, two of which are in carbonyl groups (C=O), no nitrogen or sulfur atoms, two methyl groups, and one single methylene site at the center of the molecule between the two carbonyl sites (Figure 1b). Either one of the ketones in the O=C–CH_2_–C=O site can also be in the enol form, O=C–CH–C–OH, which extends the degree of conjugation but does not change the chemical composition of curcumin/turmeric.

Both chemicals contain substituted aromatic compounds. In metanil yellow, the three aromatic rings (I, II, and III) are not identical: I is monosubstituted; II is di (1,4-)-substituted, and III is di (1,3-)-substituted. In Curcumin/Turmeric, the two rings (A and B) are structurally identical and are tri (1,3,4-)-substituted.

**Figure 2:**
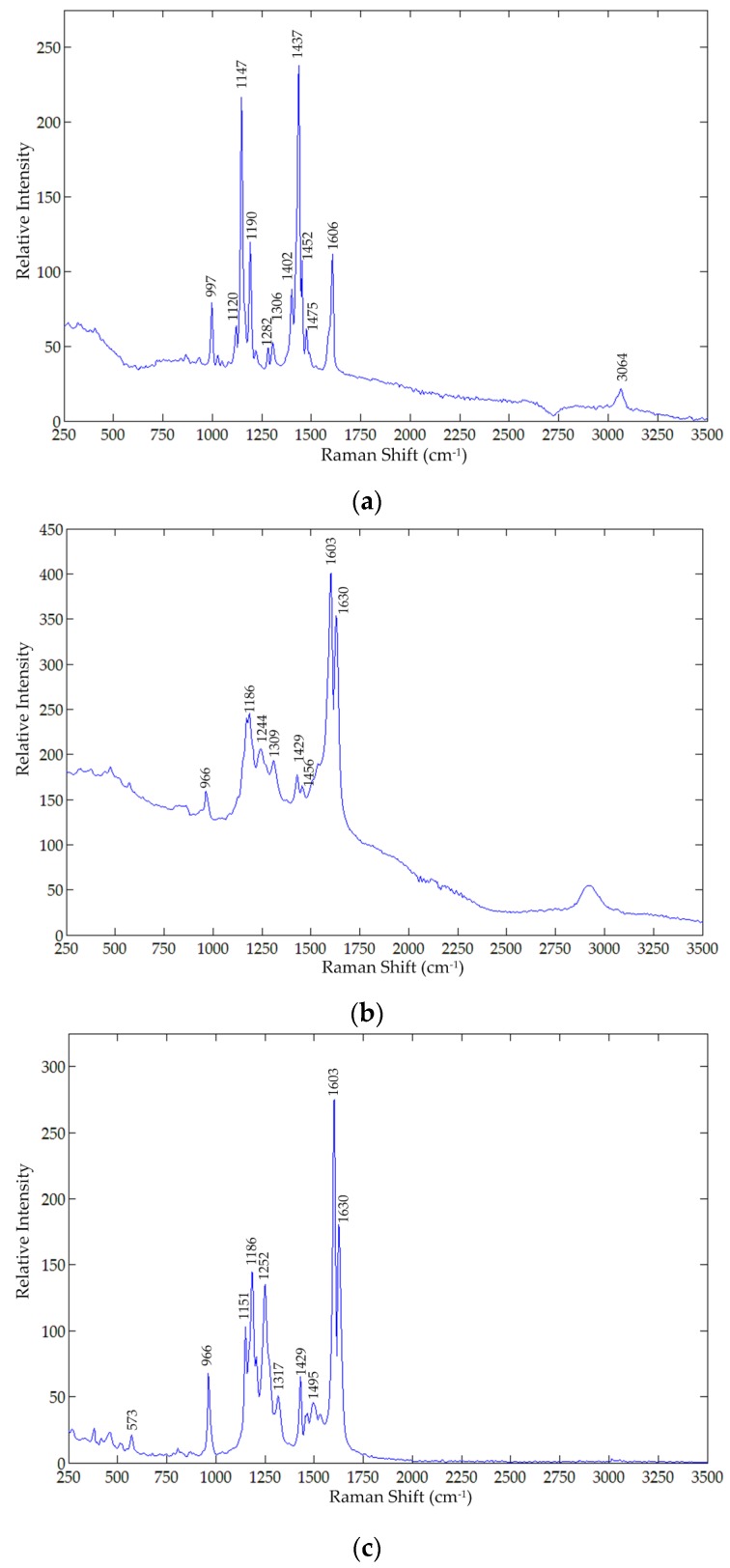
FT-Raman spectra of: (**a**) Metanil yellow; (**b**) Turmeric powder; (**c**) Curcumin.

**Figure 3:**
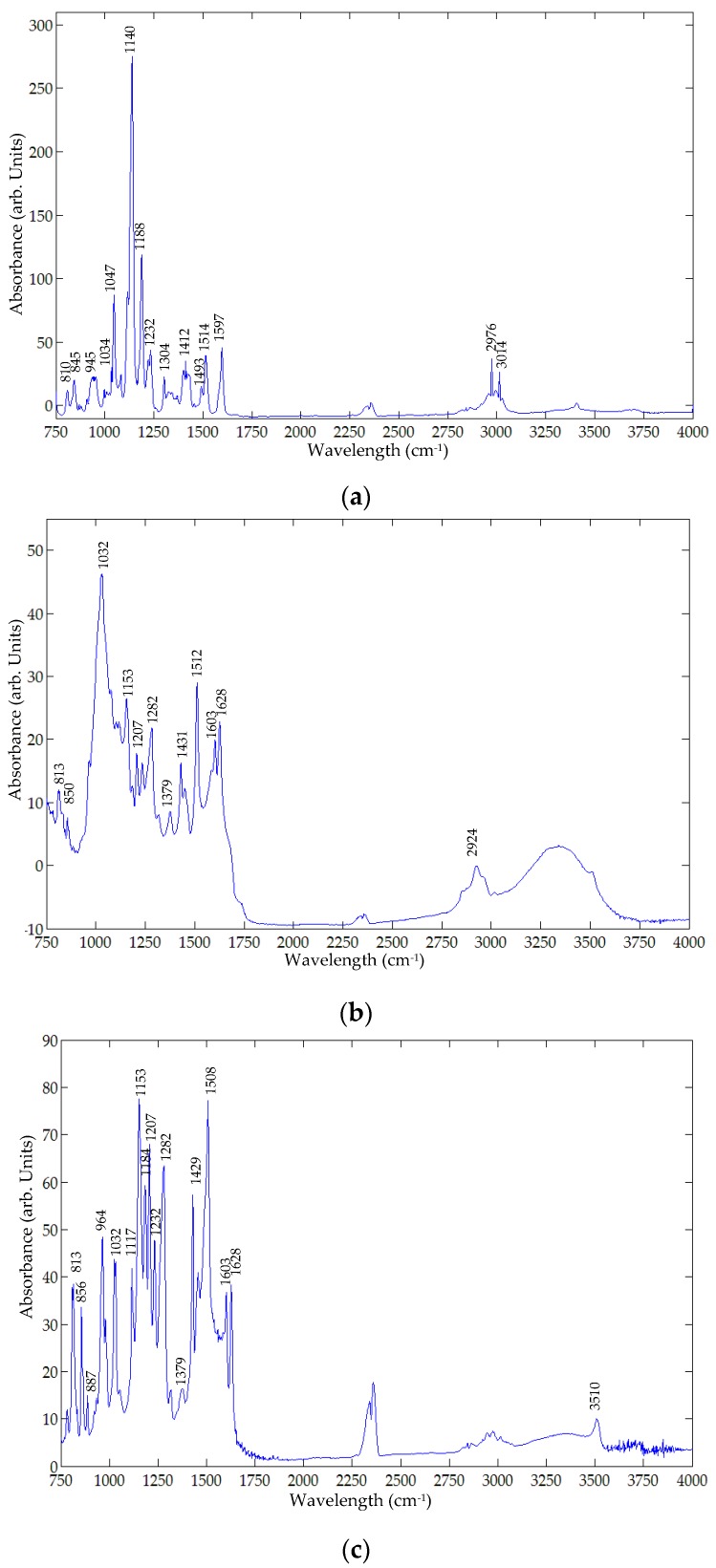
Fourier Transform-Infra Red (FT-IR) spectra of: (**a**) Metanil yellow; (**b**) Turmeric powder; (**c**) Curcumin.

Because the chemical structure of the two compounds are so dissimilar, the vibrational modes specific to precisely their discretely different chemical structures will be different. Figure 2 and Figure 3 show the FT-Raman and FT-IR spectra, respectively, of metanil yellow, turmeric, and curcumin powder (Aldrich, Carson City, NV, USA). Table 1 presents the vibrational modes of metanil yellow and turmeric in the Infrared (IR) and Raman frequency range. The assignment of structural components to vibrational frequencies is critical in this study because structurally different components can coincidentally have vibrational modes that appear in close or even the same frequency ranges. Experimental evidence is required to assign the spectral regions and their relative intensities, and to determine which intensities co-vary with concentration. The experimental spectral regions most unique to each component, and preferably with the highest relative intensity, can be used to fingerprint the presence of metanil yellow in curcumin/turmeric. Because of the abundance of spectral information collected in this experiment, further analysis of the data already collected can be used as confirmatory evidence in the fingerprint data.

In metanil yellow, sharp vibrational modes specific to the N=N site (IR 1597 cm^−1^, 1140 cm^−1^; Raman 1606 cm^−1^, 1452 cm^−1^, 1437 cm^−1^, and 1147 cm^−1^) [62,63,64] are most definitive to its identification and quantitation. Additionally, peaks due to ring breathing in ring II (IR and Raman 997 cm^−1^), and sulfate (IR 1043 cm^−1^) [65] are confirmatory of metanil yellow presence. Because metanil yellow lacks any CH_3_ or CH_2_ sites, peaks near 3000 cm^−1^ (IR 3014 cm^−1^ and 2976 cm^−1^) would be CH sites, but assigning the peaks to any specific CH site would be far from definitive. The site that results in the unique CH bending peak (IR 1188 cm^−1^ and Raman 1190 cm^−1^), however, most likely correlates to one of these CH stretching frequencies. Peaks above 3100 cm^−1^ are too broad, and often moisture dependent as well, to be analytically useful. The CH bending vibrational modes on aromatic sites below 950 cm^−1^ will be different between metanil yellow and curcumin/turmeric and could be confirmative evidence of its presence.

In turmeric, vibrational modes between 1740 cm^−1^ and 1628 cm^−1^ cannot be present in metanil yellow because the carbonyl group and conjugated carbonyl groups are only present in turmeric components. An example where structurally dissimilar sites can have similar vibrational modes is methyl deformation (IR 1187 cm^−1^, Raman 1186 cm^−1^) *versus* C–H bending in metanil yellow (IR 1188 cm^−1^, Raman 1190 cm^−1^). Two other vibrational modes fully discrete from metanil yellow are from aromatic (Ar) bending at Ar–O and Ar–O (CH_3_) (Raman 1534 cm^−1^) and CH bending at O=C–CH_2_–C=O (IR 1379 cm^−1^ and Raman 1374 cm^−1^).

Curcumin is a chemically purified fraction of turmeric. Vibrational spectroscopy can identify a structural difference between curcumin and turmeric based upon which vibrational modes are no longer present in the purified fraction. Curcumin has only one vibrational mode for C=O (IR 1628 cm^−1^, Raman 1630 cm^−1^) whereas turmeric also has two other C=O peaks (IR 1739 cm^−1^ and 1681 cm^−1^). The changes in the Ar–CH vibrational modes between the two below 950 cm^−1^ in both IR and Raman spectra suggest turmeric may contain structural analogs of curcumin as well. The Ar–OH and Ar–O–CH_3_ site at 1,3,4- on both rings A and B could be a 1,2,4- at A or B, or the methyl group could be at the 3- site instead of, or even in addition to, the 4- site. Additional chemical and spectroscopic analyses would be required to confirm the identity of the other components in turmeric. Quantitative analysis is required to determine the mole ratio of components. Although the chemical structure of curcumin can be determined precisely, the ratio of curcumin to that of other components in turmeric depends on variables including botanical and horticultural factors [3,8,24,25,26,27,28,29,30,31].

### 3.2. Evaluation of Metanil Yellow in Mixture by FT-Raman

**Figure 4:**
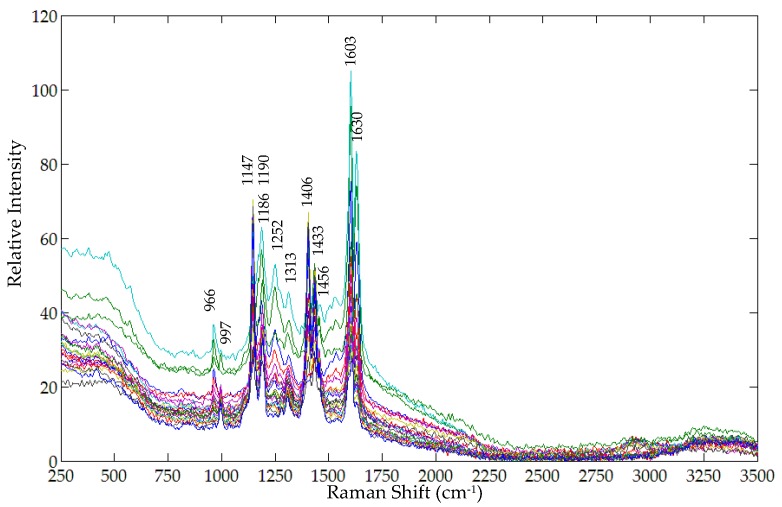
Original FT-Raman spectra of mixture samples at all concentration levels.

Figure 4 shows the original FT-Raman spectra in the 250 cm^−1^ to 3500 cm^−1^ region for all twenty-four sample mixtures (three replicates for each of eight concentrations). A majority of the FT-Raman peaks of turmeric and metanil yellow are exhibited by the mixture samples. However, the FT-Raman peaks of turmeric at 1244 cm^−1^ and of metanil yellow at 1120 cm^−1^, 1282 cm^−1^, and 1475 cm^−1^ are not seen in the mixture sample spectra at any concentration. Similarly, FT-Raman peaks of metanil yellow at 1402 cm^−1^, 1437 cm^−1^, and 1452 cm^−1^ have shifted to 1406 cm^−1^, 1433 cm^−1^, and 1452 cm^−1^, respectively, in the mixture spectra.

**Figure 5:**
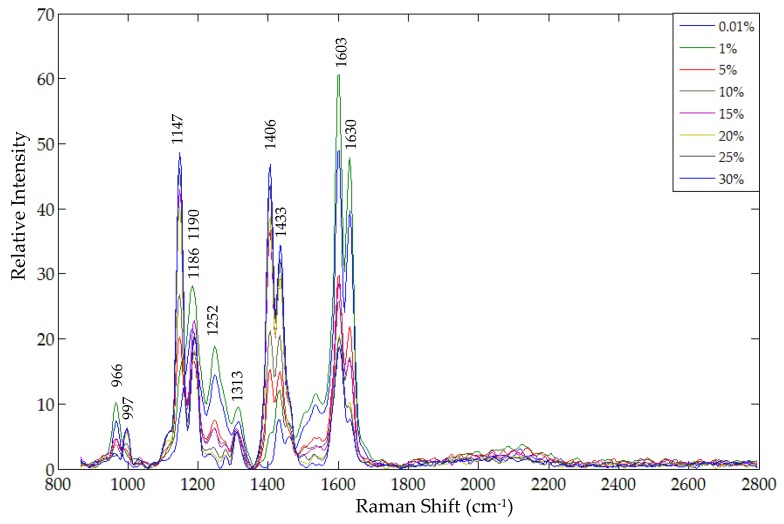
Corrected FT-Raman spectra for representative samples at each concentration level.

The FT-Raman spectra were first smoothed using a Savitzky-Golay filter (5th order polynomial curve at 3-point intervals) to remove noise. Each spectrum was then corrected to remove the underlying fluorescence background by subtracting a 6th order polynomial function fitted to the spectral fluorescence. During the spectral correction it was observed that the 6th order polynomial curve best fit the spectral region of 850 cm^−1^ to 2800 cm^−1^. Since all the spectral peaks of metanil yellow and turmeric were located in this range, only this region was utilized for spectral correction and further analysis. Figure 5 shows the corrected spectra of representative samples at each of the concentration levels.

As discussed earlier, the FT-Raman spectral peaks due to N=N (at 1606 cm^−1^, 1452 cm^−1^, 1437 cm^−1^, and 1147 cm^−1^), at 997 cm^−1^ (ring breathing in ring II), and at 1402 cm^−1^ due to S=O stretching can be utilized for identification of metanil yellow. Although each of these spectral peaks are representative peaks of metanil yellow, a few of them happen to overlap with those of turmeric powder. For example, the metanil yellow peak at 1606 cm^−1^ (due to N=N) is very close to the turmeric peak at 1603 cm^−1^ (due to C=C stretching). Similarly, the metanil yellow peak at 1437 cm^−1^ shifted to a new position at 1433 cm^−1^ (Figure 5), which is also in close proximity to the turmeric peak at 1429 cm^−1^ (Figure 2b). Because the metanil peaks at 997 cm^−1^, 1406 cm^−1^, and 1147 cm^−1^ did not overlap with any turmeric peaks, these three peaks were initially considered as representative FT-Raman peaks for metanil yellow. In addition to uniqueness or non-overlap of each compound’s characteristic peaks, selection criteria for representative peaks also included the spectral intensity of the characteristic peaks. Although the metanil yellow peaks at 997 cm^−1^ and 1147 cm^−1^ did not overlap with those of turmeric powder, the mixture samples only exhibited these key peaks for the higher concentrations of metanil yellow (Figure 5). Consequently, the FT-Raman peak at 1406 cm^−1^ was the final selection for representing metanil yellow. The intensity at 1406 cm^−1^ is high for samples with high concentrations of metanil yellow and the intensity reduces gradually with decreasing concentration, showing a relative relationship between peak intensity and metanil yellow concentration in the samples. The minimum concentration of metanil yellow for which the peak at 1406 cm^−1^ was detectable was 1%.

**Figure 6:**
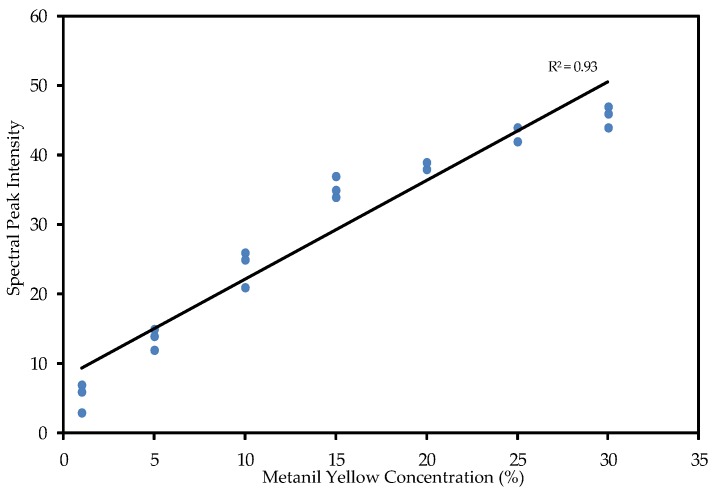
Linear relation between FT-Raman spectral peak intensity and corresponding sample concentration.

The spectral peak position is utilized to identify and distinguish chemicals in a mixture sample, while the intensity of the spectral signal can be utilized to evaluate the chemical concentration in the mixture. Two models, one based on spectral peak intensity at 1406 cm^−1^ and the other on the ratio of the peak intensity against intensity at a neutral band, were developed for evaluating metanil yellow concentration in turmeric powder. The first model evaluated the intensity of the metanil yellow peak at 1406 cm^−1^ after fluorescence removal against the metanil yellow concentrations of the samples. Figure 6 shows the relation between the metanil yellow’s peak intensity and its actual concentration for samples at seven different concentrations, with a correlation coefficient of 0.93.

For the second model, a neutral FT-Raman band—one without any sharp peaks from metanil yellow or turmeric powder—was selected for use with the metanil yellow peak at 1406 cm^−1^ in a band ratio calculation. Several neutral bands were tested for band ratio calculation. For the mixture samples, the ratio of the intensities at these two bands was correlated to actual metanil yellow concentration with a much lower correlation coefficient.

### 3.3. Evaluation of Metanil Yellow in Mixture by FT-IR

**Figure 7:**
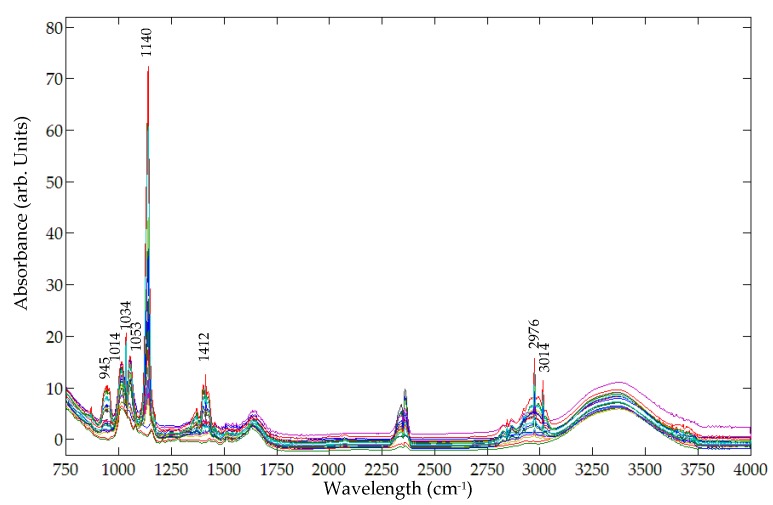
Original FT-IR spectra of mixture samples for all concentration levels.

A Nicolet 6700 FT-IR spectrometer was utilized to acquire FT-IR spectra in the spectral range of 650 cm^−1^ to 4000 cm^−1^. Figure 7 shows the original FT-IR sample spectra, each acquired as an average of 32 successive scans. Both metanil yellow and turmeric exhibit FT-IR peaks that may be utilized for detecting metanil yellow in mixture with turmeric. The metanil yellow peaks at 997 cm^−1^ and 1597 cm^−1^ due to the N=N site (Figure 3a) are not present in the FT-IR spectra of the sample mixtures (Figure 7). Since the peak at 1140 cm^−1^ (due to the N=N site in metanil yellow) shows high intensity in the sample spectra (Figure 7) and does not overlap with any peaks of turmeric powder (Figure 3b), this FT-IR peak was selected for identifying metanil yellow in the mixture sample.

**Figure 8:**
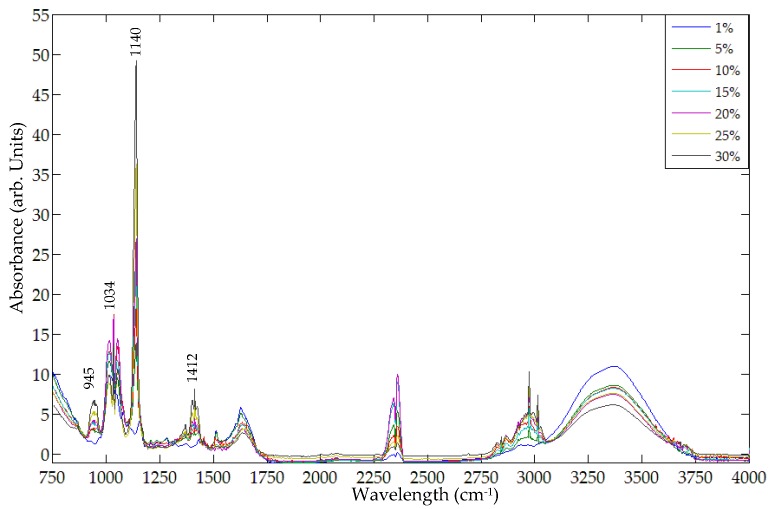
Representative FT-IR spectra of mixture samples at all concentration levels.

Figure 8 shows the representative FT-IR spectra of mixture samples at seven different concentration levels after noise removal using a Savitzky-Golay filter. The spectral peak at 1140 cm^−1^ is readily visible for sample concentrations from 30% to 5%, but vanishes at 1%. This illustrates that the 1140 cm^−1^ peak can be used to detect metanil yellow in turmeric as low as 5%.

The relation between the 1140 cm^−1^ peak intensity and metanil yellow concentration was evaluated. For all sample spectra, baseline drift was removed using the Multiplicative Scatter Correction (MSC) method. The spectral signal after baseline drift removal was utilized to develop concentration evaluation models.

**Figure 9:**
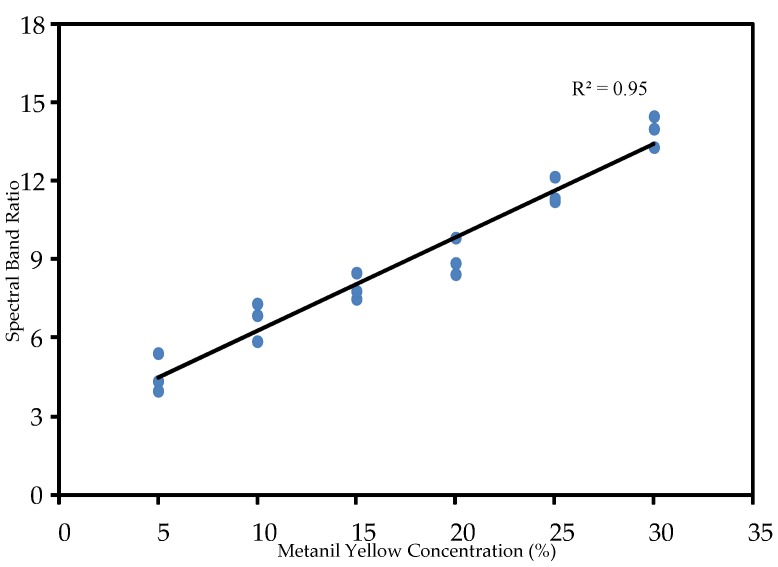
Linear relation between FT-IR spectral peak intensity and sample concentration.

Similar to the FT-Raman analysis, two models were developed for evaluation of metanil yellow concentration using the FT-IR spectra. The first model correlated the spectral peak intensity at 1140 cm^−1^ with actual metanil yellow concentrations. The result shows that the spectral peak intensity at 1140 cm^−1^ is highly correlated with metanil yellow concentration (correlation coefficient of 0.88). The second model was developed using spectral band ratio. The spectral band ratio was obtained using the spectral peak intensity at 1140 cm^−1^ and the spectral intensity of a neutrally positioned spectral band—one where the spectra are flat and lack sharp peaks for metanil yellow and for turmeric or its other constituents. Several such bands were selected and tested. The best result was obtained using the band ratio of 1140 cm^−1^ against 966 cm^−1^. It can be seen in the Figure 3a,b and Figure 8 that the spectral band at 966 cm^−1^ does not represent any chemical in the sample and has no sharp peaks. The ratio was correlated with the actual concentration of metanil yellow in the sample mixtures. The correlation coefficient of the band ratio method was found to be 0.95, higher than that for the spectral peak intensity method, as shown in Figure 9.

## 4. Conclusions

This study utilized FT-Raman spectroscopy and FT-IR spectroscopy techniques for the detection of metanil yellow in turmeric powder. FT-Raman and FT-IR spectra of metanil yellow, turmeric, and curcumin were acquired and analyzed. The FT-Raman spectral peak at 1406 cm^−1^ and FT-IR spectral peak at 1140 cm^−1^ were selected as representative peaks for metanil yellow. FT-Raman and FT-IR spectral analysis of sample mixtures of metanil yellow in turmeric at eight different concentrations showed that the FT-Raman method was able to detect metanil yellow at 1% concentration, while detection by the FT-IR method was limited to 5% concentration. For both FT-IR and FT-Raman analysis, two models were developed to evaluate metanil yellow concentration. The first model simply correlated a single peak intensity (FT-Raman 1106 cm^−1^ and FT-IR 1140 cm^−1^) to the actual sample concentration. The second model used a ratio of peak intensity and intensity at a neutral band. The band ratio model performed better for the FT-IR analysis (correlation coefficient of 0.95), while the peak intensity model was superior (correlation coefficient of 0.93) for the FT-Raman analysis. The results show that FT-Raman and FT-IR spectroscopy can be utilized for detection of chemical contaminants in food powders such as carcinogenic metanil yellow in turmeric.

## Figures and Tables

**Table 1:** Assignment of FT-Raman and FT-IR spectral bands [62,63,64,65].

Metanil Yellow			Turmeric		
Infra Red (IR) (cm^−1^)	Raman (cm^−1^)	Assignment	IR (cm^−1^)	Raman (cm^−1^)	Assignment
			3342 br		O–H str intermolecular bonded
	3064	ν (C–H)		3073	O–H str alcohol
3030	3043	ν(C–H)			
**3014** sharp			3017		
**2976** sharp				2974	
2956			2959		
2923 weak	2926 weak		2924		
			2874		
			2854	2855	
			1739		C=O stretching
			1681		Conjugated C=O stretching
			1628	1630	Disubstituted C=C stretching
**1597** sharp	**1606** sharp	**ν(N–N) stretching (III)**	1603	1603	C=C stretching
1581	1591	ν(C–C) stretching (III)	1585		
				1534	δ(Ar–O + Ar–O–R) bending
**1514**	1524 weak	ν(C–C) stretching (III)	1512		
				1505	
1493	1496 weak	ν(C–C) stretching (I)			
	1475	ν(N=N), δCH	1465		CH bending
1455 weak	**1452** sharp	**ν(N=N), δCH**	1451	1456	
1431	**1437** sharp	**ν(N=N)**	1431	1429	
1412	1417	ν(N=N) stretching (I)			
1400	1402	S=O str			
			1379	1374	C–H bending in O=C–CH_2_–C=O
1371		S=O str			
1343		S=O str			
1325		ν_as_(SO_2_)	1318		
1304	1306	νC–C stretching (III)		1309	
	1282	νC–N stretching	1282		
1264 weak		ν(C–N_azo_)δ(C–H)		1268	COH + CO(CH_3_) stretching
1232	1243 weak	νC–X stretching (I)	1234	1244	CH bending
1223	1218 weak	δ(N–H)			
			1207	1202	C–O–(CH_3_) stretching
**1188**	**1190**	**δ(C–H)**	1187	1186	CH_3_ deformation
1171		ν(C–N_azo_)δ(C–H)		1173	CH bending
				1156	
**1140** sharp	**1147** sharp	**ν(C–N_azo_)δ(C–H)**			
**1115** sharp	1120		1125	1126	
1082	1084 weak	βCH bending (II) ip	1076	1084	
**1047**	1052 weak			1045	
1034		ν_s_(SO_3_)	1032	1028	C–O stretching
1023	1026 weak				
997	**997** sharp	**Ring breathing (II)**			
			969	966	=CH wag trans
945					
936	935 weak		930	938	
908		γCH wagging (I,II) op		901	
882 weak		γCH wagging (II) op	888		Ar CH bending
870 weak	866 weak	γCH wagging (I) op	870	860	Ar CH bending
845	842 weak	γCH wagging (II) op	856		Ar CH bending
830		γCH wagging (III) op	837		
810	813 weak	1,4-Ar CH bending	813	812	Ar CH bending
